# Effect of educational intervention in reducing exposure to second hand tobacco smoke among 12-year-old children as determined by their salivary cotinine levels and knowledge, attitude and behavior - a randomized controlled trial

**DOI:** 10.3389/froh.2023.1277307

**Published:** 2023-09-28

**Authors:** Ashwini Rao, Nikita Rungta, M. Nandini, B. Unnikrishnan, Ramya Shenoy, Arathi Rao, Mranali K. Shetty

**Affiliations:** ^1^ Department of Public Health Dentistry, Manipal College of Dental Sciences, Mangalore, Manipal Academy of Higher Education (MAHE), Manipal, India; ^2^ University of Michigan School of Dentistry, Ann Arbor, MI, United States; ^3^ Department of Biochemistry, Kasturba Medical College, Mangalore, Manipal Academy of Higher Education (MAHE), Manipal, India; ^4^ Department of Community Medicine, Kasturba Medical College, Mangalore, Manipal Academy of Higher Education (MAHE), Manipal, India; ^5^ Department of Pedodontics and Preventive Dentistry, Manipal College of Dental Sciences, Mangalore, Manipal Academy of Higher Education (MAHE), Manipal, India; ^6^ Department of Periodontics, Manipal College of Dental Sciences, Mangalore, Manipal Academy of Higher Education (MAHE), Manipal, India

**Keywords:** adolescent, cotinine, environmental exposure, health education, randomized controlled trial, saliva, second-hand smoke, tobacco

## Abstract

**Background:**

Tobacco use is one of the most important public health concerns, with approximately 8.7 million tobacco-related deaths each year, primarily in low- and middle-income countries. Even more concerning is the fact that 1.3 million of these deaths are seen in nonsmokers, including babies and children. This study was performed to determine whether a school-based “tobacco-free” educational intervention program among 12-year-old children would be effective in reducing their exposure to second-hand tobacco smoke (SHS) by improving their knowledge, attitude and behavior post intervention and estimating salivary cotinine levels as markers of SHS exposure.

**Materials and method:**

A randomized controlled trial was performed by a cluster random sampling technique, with 30 participants each in the experimental and control arms. A knowledge, attitude, avoidance behavior and self-efficacy of avoidance questionnaire was administered, followed by estimation of salivary cotinine levels. The experimental arm received the “tobacco-free” intervention, which comprised a 40-min health education session, with the first follow-up at 15 days and the second at 30 days after the intervention. After the intervention, the questionnaire was readministered, followed by re-estimation of salivary cotinine levels.

**Results:**

One month after the intervention, the number of participants who had a smoker who lived with them and the number of people who smoked inside the house were reduced in the experimental group compared to the control group. In the knowledge domain and the attitude domain, 80% and 60% of the items showed a statistically significant improvement in the experimental group compared to the control group. In the avoidance behavior domain and the Self-Efficacy of Avoidance Domain, all the items showed improvement in the experimental group compared to the control group. When the mean salivary cotinine levels were compared pre- and postintervention, it was found that although the mean postintervention salivary cotinine levels increased in both the experimental and control groups, the increase was less in the experimental group than in the control group.

**Conclusion:**

The present study has been shown to be effective in improving the knowledge, attitude and avoidance behavior of adolescents toward exposure to secondhand smoke.

## 1. Introduction

“*The scientific evidence is clear: there is no safe level of second-hand smoke* (1)”

Tobacco use is one of the most important public health concerns, with approximately 8.7 million tobacco-related deaths each year, primarily in low- and middle-income countries. Even more concerning is the fact that 1.3 million of these deaths are seen in nonsmokers, including babies and children. Almost half of all children are exposed to tobacco-polluted air, and 65,000 children die each year as a result of illnesses induced by second-hand smoke (1).

It has been reported that in India, 11% of 13- to 15-year-olds are exposed to SHS at home, and 21% are exposed to SHS in enclosed public spaces (2). Second-hand smoke (SHS) is a mixture of compounds produced by tobacco ingested by an “active smoker” and is often referred to as “passive smoke”, “environmental tobacco smoke (ETS)”, or “tobacco air pollution”. At least 69 of the 7,000 chemicals emitted could cause cancer (1). SHS includes both side-stream smoke from the end of the cigarette and smoke exhaled by smokers. Smoke from one room flows to other rooms in the building regardless of whether doors are kept locked or windows are kept open. SHS can linger indoors for hours and become increasingly hazardous over time (3). Toxic components of SHS stick to rugs, draperies, clothing, food, and other surfaces and stay in the room for months after active smoking has stopped, resulting in “third-hand smoke” (THS), which is identified as an SHS side effect (4). THS, also known as “residual tobacco smoke” or “aged tobacco smoke”, can harm anyone who is exposed to it.

In adults, SHS exposure has been associated with stroke, coronary heart disease, cancer, chronic obstructive pulmonary disease, respiratory infections, and other illnesses, while in children, it has been linked to severe asthma, respiratory tract infections, ear infections, and sudden infant death syndrome (5–7).

Because their lungs and bodies are still developing and they breathe faster than adults, young children are more vulnerable. Furthermore, passive smoking is detrimental to mental health, may cause depressive symptoms (3, 8) and diminishes cognitive performance (9).

Although legislation banning smoking in enclosed public places has been widely introduced, the global progress on implementation remains uneven, with implementation rates ranging from 13% to 85% (10). Many laws have been enacted in India to discourage tobacco consumption, beginning with the Cigarettes Act of 1975. In 2003, the Cigarette and Other Tobacco Products Act was passed, and the Framework Convention on Tobacco Control came into effect in 2005. In 2008, the Government of India passed legislation prohibiting smoking in public places (11, 12). These laws have given the general public some optimism that the tobacco epidemic may one day come to an end.

However, none of these laws prohibit smoking inside households, and it has been discovered that children of smoking parents are especially vulnerable to SHS exposure at home since children spend so much time with their parents (13, 14).

Some studies (15, 16) have shown that interventions targeted at parents for bringing about smoking cessation/reduction, thereby reducing ETS at home, improved child health outcomes and reduced not only the risk of tobacco exposure among children but also reduced initiation of the smoking habit. However, systematic reviews have shown that the effectiveness of these interventions was not clearly demonstrated (17), and even if outcomes were present, they were not sustained for a long period of time (18, 19).

This brings us to our research question, i.e., can educational programs targeted at children bring about an improvement in their knowledge, attitude and behavior toward SHS, thus reducing their exposure to the same. A literature search revealed no evidence pertaining to targeted intervention programs affecting children. This led to the conceptualization of the present study, with the aim of assessing the effect of a “tobacco-free” educational intervention among 12-year-old children in reducing their exposure to second-hand tobacco smoke. The objective was to determine their knowledge, attitude and behavior pre- and postintervention along with estimating salivary cotinine levels as markers of SHS exposure. Since salivary cotinine is a sensitive biochemical marker that is highly linked to SHS exposure and has a half-life of 16 h, it has been extensively used as a marker for detecting both active and passive smoking (20).

## 2. Materials and methods

### 2.1. Study design & study setting

This study is a component of a multiphase study that was conducted among 12-year-old school children in Mangalore, India. The first phase was a descriptive cross-sectional study that used a cluster random sampling of all schools, which is described in detail elsewhere (21). The 12-year-olds in the selected schools who met the inclusion criteria, i.e., who consented to participate in the study and those participants whose parents gave written informed consent and they themselves give informed assent, were given a questionnaire. Participants who reported second-hand cigarette smoke exposure were chosen at random, and their salivary cotinine levels were measured. All participants with salivary cotinine levels greater than 0.1 ng/ml (22) were included for sampling.

### 2.2. Sample size calculation and random allocation

G Power 3.1.2 was used to calculate the sample size. We determined a total sample size of 60, with 30 in the control group and 30 in the experimental group, 90% power, an effect size of 0.3, a 95% confidence interval, and a 10% attrition rate. Since no studies were available in this age group to obtain the standardized mean difference in salivary cotinine values between the two groups, we assumed a Cohen's *d* of 0.3, since it is generally agreed that the means of the two groups should differ by at least 0.3 standard deviations to say that the difference is significant.

#### 2.2.1. Criteria for inclusion

**Figure 1:**
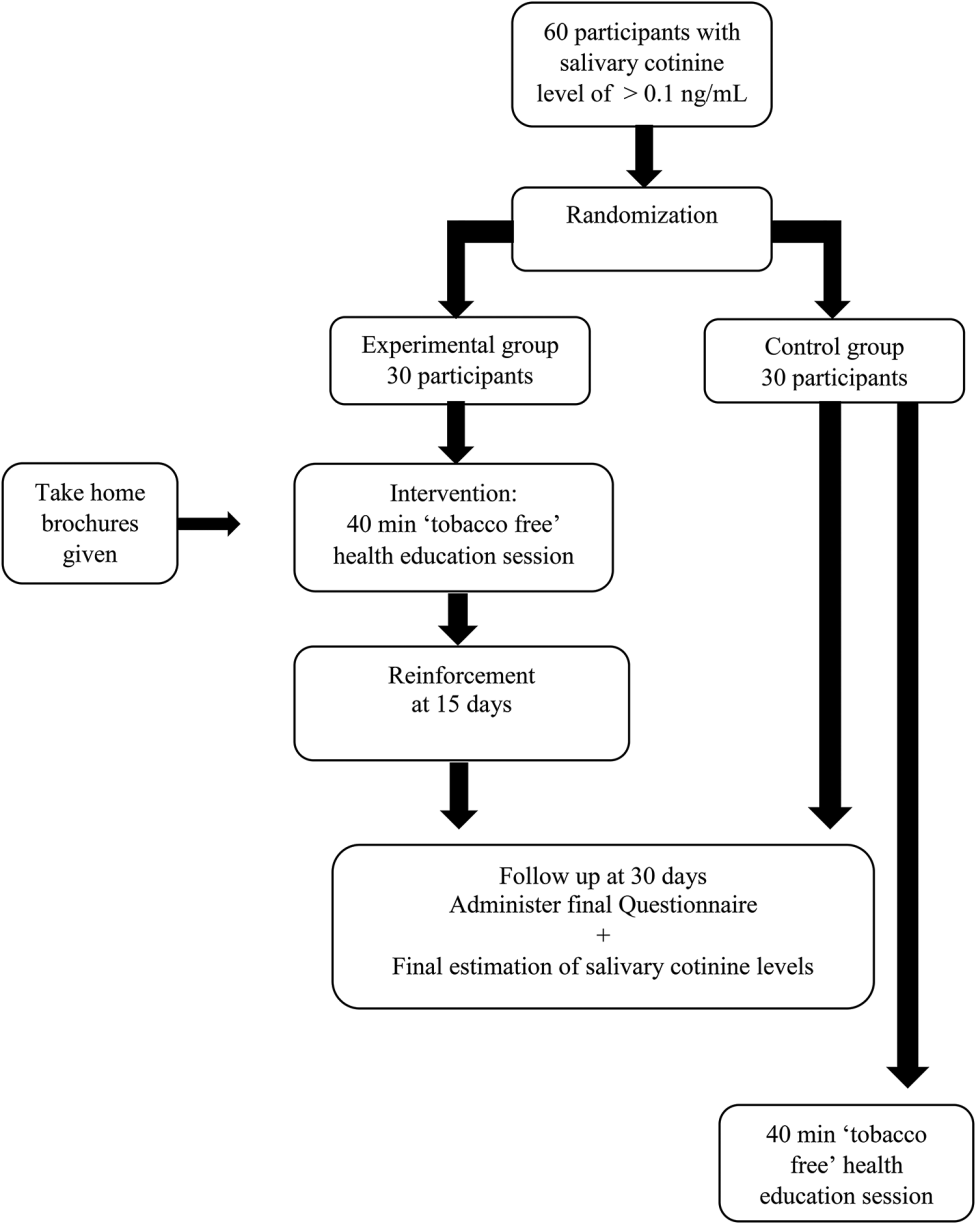
Flowchart of participants.

All participants with salivary cotinine levels greater than 0.1 ng/ml (20) were grouped school wise, and the school clusters were included in the sampling frame. Clusters of schools were chosen instead of individual participants to eliminate contamination bias that could occur if both experimental and control groups were present in the same school. Cluster random sampling was then performed for the current randomized controlled trial (Figure 1).

Random allocation: Allocation concealment was accomplished through the use of sequentially numbered, opaque, sealed envelopes (SNOSE), and clusters were divided into experimental and control groups by simple randomization. We chose one school to be the experimental group and another to be the control group. The outcome assessors were blinded in the trial, and the random allocation process was carried out by one of the authors (SM).

### 2.3. Data collection

#### 2.3.1. Questionnaire

The questionnaire was in the English language and comprised three components: the first was demographic data, and the second was a 5-item assessment of children's exposure to SHS (23). The third component was the 25-item questionnaire (24) designed to assess children's secondhand smoke knowledge, attitude, avoidance behavior, and self-efficacy of avoidance. Out of the 25 items, ten items measured knowledge, five items assessed attitude, five items assessed avoidance behavior against secondhand smoke, and five items assessed self-efficacy of avoidance. More details are given elsewhere (21). The questionnaire assessment was performed twice, once at baseline and once at one month after the intervention.

Pilot study: Content validity was assessed by subject experts (UB and RS). The questionnaire was administered to ten twelve-year-old children who were not part of the study, and a Cronbach's alpha of 0.8 was obtained, showing high reliability.

#### 2.3.2. Salivary cotinine estimation

Salivary cotinine is undetectable in people who do not smoke and in those who are not exposed to passive smoking. A saliva cotinine concentration of less than 10 ng/ml suggests potential passive exposure, while a concentration greater than 10 ng/ml indicates active nicotine usage (20).

Saliva collection: Saliva collection was performed twice during the course of the study, once at baseline before the intervention and the other at 30 days after the intervention. Saliva collection from participants was scheduled in the mornings, approximately one hour after their morning breakfast. They were asked to rinse their mouth thoroughly with water 10 min before saliva collection. Labelled saliva collection boxes were distributed. They were then asked to tilt their head forward to allow the saliva to pool in the floor of the mouth. Whole saliva was collected by unstimulated passive drool. Collected saliva samples were immediately placed in an ice chest and transported to the laboratory within 30 min. The pH of all the samples was noted using Fisher Scientific Indikrom Papers. Samples with a pH less than 3.5 or more than 9.0 were discarded and recollected the next day. The samples were refrigerated before analysis.

#### 2.3.3. Laboratory analysis

The laboratory analysis was performed by an expert biochemist (NM). On the day of centrifugation, the samples were transferred into labelled centrifugation tubes and centrifuged at 5,200 rpm for 15 min. The clear supernatant was pipetted out, and the centrifuge was stored in Eppendorf tubes at −21°C until analysis. The samples were allowed to thaw completely before carrying out the assay. The Salimetrics® High Sensitivity Salivary Cotinine Quantitative Enzyme Immunoassay Kit, a competitive immunoassay kit, was used for the assay (25).

#### 2.3.4. Reagent preparation

All reagents were brought to room temperature and mixed before use. The microtiter plate was also brought to room temperature while inside the foil pouch to keep it away from moisture contamination. A 1× wash buffer was prepared as per the kit insert. Serial dilutions of cotinine standards were prepared, and the final concentrations achieved were 200 ng/ml, 66.7 ng/ml, 22.2 ng/ml, 7.4 ng/ml, 2.5 ng/ml and 0.8 ng/ml. After the reagents were ready, the plate layout was determined. The microplate incubator/shaker was set to 37°C.

#### 2.3.5. Sample preparation

Twenty microliters of standards, controls, and saliva samples were pipetted into appropriate wells. Twenty microliters of assay diluent was pipetted into 2 wells to serve as the zero. For nonspecific binding (NSB) wells, 120 μl of assay diluent was pipetted. The diluted enzyme conjugate solution (1:300) was immediately mixed, and 100 μl was added to each well using a multichannel pipette. One hundred microliters of cotinine antiserum was pipetted into all wells, except the NSB wells, using a multichannel pipette.

The plate was covered with an adhesive cover and was constantly mixed at 500 rpm in a preheated 37°C microplate incubator/shaker for 0.5 h at 37°C. The plate was then washed 4 times with 1× wash buffer by pipetting 300 μl of wash buffer into each well and then discarding the liquid. Before turning the plate upright, it was thoroughly blotted on paper towels. A multichannel pipette was used to add 200 μl of the TMB (3,3′,5,5′-tetramethylbenzidine) substrate solution to each well. Before incubating in the dark at room temperature for 25 min, the plate was mixed on a plate rotator for 5 min at 500 rpm. A multichannel pipette was used to add 50 μl of stop solution, which was then mixed on a plate rotator for 3 min at 500 rpm. At this stage, the green color turns to yellow in all the wells. The bottom of the plate was dried by wiping off with a water-moistened, lint-free cloth. An ELISA plate reader (Lisa plus microplate reader) was used to read the plate at 450 nm and 620 nm within 10 min of adding the stop solution.

The “High” and “Low” Salimetric controls were run with each assay. The concentrations of the controls and the samples were determined by interpolation using data reduction software (4-parameter nonlinear regression curve fit). The sensitivity of the assay was 0.15 ng/ml.

#### 2.3.6. Intervention

The intervention was carried out by the principal investigator (RA) and consisted of a 40-min health education session addressing tobacco and its adverse effects, laws regarding tobacco in India and the world and information on how to prevent exposure to tobacco smoke. Children were also given posters that contained messages on the effects of tobacco and how to make their homes smoke-free, to be placed in their homes.

The health education intervention was delivered at baseline, and the children were recalled 15 days after the intervention to reinforce the health education session and to discuss their experiences and the challenges they faced.

Participants in the control arm received conventional standard health education once at the end of the study.

### 2.4. Statistical analysis

It was done based on the Intention to Treat analysis. The data were entered into SPSS software (IBM Corp. Released 2011. IBM SPSS Statistics for Windows, Version 20.0. Armonk, NY: IBM Corp.) and analysed. Descriptive statistics are presented, and subgroup analysis was performed wherever needed. The level of significance was kept at 0.05 with 95% confidence levels. The chi-square test was performed for categorical variables, and the paired t test was performed to compare means.

### 2.5. Ethical considerations

Ethical approval was obtained from the Institutional Ethics Committee (Ref No. 17021). Written informed consent from parents and informed assent from the participants was obtained prior to recruitment. There were no personal identifiers on the vials sent to the laboratory, and individual results have not been disclosed. Confidentiality of the participants was maintained at all times. The saliva collected was completely utilized for laboratory evaluation, and no biological specimens were stored for further use. This trial has been registered with the Clinical Trial Registry of India (CTRI—09/015706).

The article follows the CONSORT 2010 checklist when reporting a cluster randomized trial (Annexure 1).

## 3. Results

In the phase 1 part of the study (21), 236 participants reported that somebody who lived with them smoked tobacco. Among them, 142 were willing to participate in this phase of the study. The saliva samples of these 142 children were collected and analysed. Of them, 112 were found to have a salivary cotinine level of more than 0.1 ng/ml, and 30 had less than 0.1 ng/ml. We obtained 3 school clusters that had at least 30 participants who could participate in this study. Two schools were randomly allocated, one each into the experimental and control arms. From each school, 30 participants were randomly selected to obtain our sample size of 60 participants, which comprised a total of 33 boys and 27 girls. There was no attrition of study participants, and the originally assigned groups were analysed.

### 3.1. Exposure to SHS

When we analysed the responses of the groups based on the correct responses for the questionnaire, at baseline, we found that all 60 participants reported being exposed to tobacco smoke at home. The children also reported that most of the smokers smoked inside the house, 80% in the experimental group and 93.3% in the control group. Thirteen children from the experimental group reported that people who visited their house smoked, although 12 of them said that they smoked outside, and only one reported that they smoked inside the house. In the control group, among the 12 students who reported that people who visited their house smoked, 7 of them said that they smoked outside, whereas 5 reported that they smoked inside the house. Among the experimental group, only 2 children reported that people who lived with them smoked in front of children, whereas in the control group, 6 did.

When we compared the baseline and final scores of the experimental and control groups, we found that at baseline, although all participants, both in the experimental and control groups, had a smoker who lived with them, one month after the intervention, we found that 22 participants in the experimental group and 20 participants in the control group reported that the smoker who lived with them had stopped smoking. Even the number of smokers who visited their house was reduced in both groups. We also found that 24 people smoked inside the house at baseline in the experimental group, which was reduced to 5 after the intervention (Table 1).

**Table 1:** Comparison of baseline and final scores based on exposure to SHS.

	Experimental group		Control group	
	Baseline	Final	Baseline	Final
Someone else you live with smokes	30	8	30	10
People who visit your home, smoke	13	10	12	10
People who stay with you, smoke inside the house	24	5	28	7
People who stay with you, smoke outside the house	6	6	2	2
People who visit your home, smoke inside the house	1	1	7*	1
People who visit your home, smoke outside the house	12*	9	5	9
People who smoke in front of children	2	4	6	6

* *P* value: 0.03; chi square value 13.989.

However, the numbers of those who smoked outside the house remained the same in both groups. After the intervention, the number of house guests who smoked outside decreased in the experimental group but increased in the control group. This was found to be statistically significant (*P* = 0.03). A bewildering finding was that more children reported that the number of people who smoked in front of children increased after the intervention in the experimental group but remained unchanged in the control group. This was also found to be statistically significant (Table 1).

### 3.2. Questionnaire

#### 3.2.1. Knowledge domain

Both in the control group and in the experimental group, more participants had good knowledge that a current smoker's child has a higher risk for developing lung cancer than a nonsmoker. Many participants were also aware that long-term second-hand tobacco smoke was responsible for lung cancer in nonsmokers. When compared with baseline, the number of participants who understood that secondhand tobacco smoke was generated from the burning end of a cigarette or from the cigarette smoke puffed out by smokers increased in the experimental group and decreased in the control group. This was found to be statistically significant (*P* = 0.037).

The number of participants who knew that a smoldering cigarette was more toxic than the smoke exhaled by a smoker increased after intervention in the experimental group but remained the same in the control group (*P* = 0.003). The number of participants who knew that even if not actively smoking, they had to worry about the damage to their health that may be caused by secondhand tobacco smoke increased from 15 to 23 in the experimental group but remained unchanged at 18 in the control group, and this increase was found to be statistically significant (*P* = 0.05). However, the participants who knew that a lit cigarette burning in an ashtray would affect the health of people nearby increased statistically both in the experimental and in the control group (*P* = 0.027). Although only 8 participants in the experimental group at baseline knew that long-term secondhand tobacco smoke affected the lungs and the heart, it increased to 24 after the intervention. This was found to be statistically significant (*P* = 0.000) when compared to the control group. Similarly, the number of participants who knew that not only train and airplane passengers but also car passengers should not smoke was found to statistically increase after the intervention (*P* = 0.001) (Table 2).

**Table 2:** Comparison of baseline and final scores based on knowledge (weighted chi-square).

	Experimental group		Control group		Chi square value	df	*P* value
	Baseline	Final	Baseline	Final			
Second-hand tobacco smoke is generated from the burning end of a cigarette or from the cigarette smoke puffed out by smokers	8	11	18	16	8.482	3	0.037^*^
Even though I do not smoke, long-term exposure to second-hand tobacco smoke will be harmful to my health	18	23	16	20	3.878	3	0.275
A smoldering cigarette is more toxic than the smoke that is exhaled by a smoker	11	25	18	18	13.611	3	0.003^*^
Even if not actively smoking, one has to worry about the damage to one's health that may be caused from second-hand tobacco smoke.	15	23	18	18	12.603	3	0.05
If one is a current smoker, one's child has a higher risk for developing lung cancer	24	20	22	25	2.683	3	0.443
A lit cigarette burning in an ashtray will affect the health of people nearby	13	22	17	23	9.209	3	0.027^*^
Long-term second-hand tobacco smoke affects the lungs and the heart	8	24	22	21	22.578	3	0.000^*^
Long-term second-hand tobacco smoke is responsible for lung cancer in nonsmokers.	22	20	21	21	0.371	3	0.957
Not only train and airplane passengers, but even car passengers cannot smoke	13	24	19	26	15.558	3	0.001^*^
Second-hand tobacco smoke is a toxic cocktail consisting of cancer producing chemicals	8	16	16	15	6.008	3	0.111

* *P* < 0.05 = Significant.

#### 3.2.2. Attitude domain

In both groups, at baseline, 26 out of 30 felt that it was worthwhile to take the initiative to avoid passive tobacco smoke to protect one's health. We also found that the majority of the participants felt that they needed to pay constant attention to the avoidance of secondhand tobacco smoke. However, very few participants in both groups felt that when family members or friends smoke in the home, it was okay to avoid the area where they are smoking. Only 17 participants felt that whenever someone smoked beside them, it was a troublesome matter, and among them, only 2 were in the experimental group, i.e., 28 participants in the experimental group did not find second-hand smoke a troublesome matter. However, 42 participants felt that in a second-hand smoke environment, by asking smokers not to smoke, they were doing something to protect their health.

The only statistically significant difference was found in that in the experimental group, participants who felt that whenever someone smoked beside them, it was a troublesome matter increased from 2 to 5 after intervention, whereas it decreased in the control group from 15 to 8 after a month (*P* = 0.001) (Table 3).

**Table 3:** Comparison of baseline and final scores based on attitude (weighted chi-square).

	Experimental group		Control group		Chi square value	df	*P* value
	Baseline	Final	Baseline	Final			
I think it is worthwhile to take the initiative to avoid passive tobacco smoke in order to protect one's health.	26	21	26	26	4.329	3	0.228
I think we need to pay constant attention to the avoidance of second-hand tobacco smoke	26	27	28	27	0.741	3	0.864
When family members or friends smoke in the home, I think it is okay to avoid the area where they are smoking.	12	16	17	16	1.967	3	0.579
Whenever someone smokes beside me, it is a troublesome matter	2	5	15	8	16.553	3	0.001*
When you are in a second-hand smoke environment, by asking smokers not to smoke, or requesting them to smoke somewhere else, you are doing something to protect your health.	21	19	21	26	4.472	3	0.215

* *P* < 0.05 = Significant.

#### 3.2.3. Avoidance behavior domain

When we analysed avoidance behavior with respect to second-hand smoke, all 30 participants in the control group and 22 in the experimental group reported that if someone from their family smoked in front of them, they would choose to leave to avoid second-hand smoke.

The number of participants who were confident of choosing to leave if someone from their family smoked in front of them to avoid secondhand smoke increased significantly in the experimental group but decreased in the control group (*P* = 0.009). The number of participants who reported that when they could not avoid a second-hand smoke environment, they would open the window to ventilate the smoke in the room also increased after intervention (*P* = 0.000) (Table 4).

**Table 4:** Comparison of baseline and final scores based on avoidance behavior (weighted chi-square).

	Experimental group		Control group		Chi square value	df	*P* value
	Baseline	Final	Baseline	Final			
In my family, if someone smokes in front of me I will choose to leave in order to avoid the second-hand smoke	22	28	30	24	11.538	3	0.009^*^
In public places when people smoke in front of me, I will choose to leave in order to avoid the second-hand smoke	21	23	23	27	3.72	3	0.292
When I cannot avoid a second-hand smoke environment, I will open the window to ventilate the smoke in the room.	12	26	11	15	19.018	3	0.000^*^
In my home, if someone smokes in front of me I will ask him or her to stop smoking or I will ask him or her to smoke elsewhere	21	25	24	20	3.022	3	0.388
In public places, if someone smokes beside me I will ask him or her to stop smoking, or I will ask him or her to smoke elsewhere	15	23	22	16	7.177	3	0.066

* *P* < 0.05 = Significant.

#### 3.2.4. The self-efficacy of avoidance domain

Although most of the participants had the confidence to request that their friends and family members stop smoking, only 58.3% had the confidence to ask strangers not to smoke in public spaces. Approximately 67% of the participants were confident that they could avoid secondhand smoke while with relatives or elders.

After the intervention, the number of participants who had the confidence to ask family members to stop smoking at home and strangers not to smoke in banned public spaces increased from 20 to 26 (*P* = 0.021) and 18 to 23 (*P* = 0.039), respectively. After the intervention, 24 participants were confident that they could avoid secondhand smoke while with relatives or elders, compared to 15 at baseline, and this difference was found to be statistically significant (*P* = 0.014) (Table 5).

**Table 5:** Comparison of baseline and final scores based on self-efficacy of avoidance (weighted chi-square).

	Experimental group		Control group		Chi square value	df	*P* value
	Baseline	Final	Baseline	Final			
I have the confidence to request my friends to stop smoking	21	28	25	24	5.566	3	0.135
I have the confidence to request my family members to stop smoking in the home	20	26	28	27	9.693	3	0.021^*^
I have the confidence to ask strangers not to smoke in banned public spaces	18	23	17	12	8.366	3	0.039^*^
I am confident that I can avoid second-hand smoke while with friends	21	27	25	24	4.034	3	0.258
I am confident that I can avoid second-hand smoke while with relatives or elders	15	24	25	18	10.629	3	0.014^*^

* *P* < 0.05 = Significant.

When we compared the baseline domain scores with postintervention scores, we found that out of the four domains, the domains of knowledge and avoidance behavior showed statistically significant improvement in the domain scores of the experimental group (Table 6).

**Table 6:** Comparison of baseline and final domain scores.

Domain	Experimental group		Control group		Chi square value	Degree of freedom	*P* value
	Before	After	Before	After			
Knowledge	10	24	20	19	14.655	3	0.002^*^
Attitude	22	22	26	26	3.333	3	0.343
Avoidance behavior	21	26	28	21	11.462	3	0.009^*^
Self-efficacy of avoidance	22	26	25	23	7.374	3	0.061

* *P* < 0.05 = Significant.

### 3.3. Salivary cotinine levels

The mean baseline salivary cotinine levels were 0.625 in the experimental group and 0.64 in the control group.

The mean salivary cotinine levels increased in both the experimental and control groups. This increase was found to be 0.091 in the experimental group compared to 0.139 in the control group. Although this difference was not found to be statistically significant, we consider this higher increase in the salivary cotinine levels in the control group to be clinically significant (Table 7).

**Table 7:** Mean salivary cotinine levels at baseline and postintervention.

	Experimental group	Control group
Baseline	0.625 ± 0.786	0.640 ± 0.705
Final	0.716 ± 0.675	0.779 ± 0.783

*P* = 0.853.

## 4. Discussion

The purpose of this study was to determine whether a school-based “tobacco-free” educational intervention program among 12-year-old children would be effective in reducing their exposure to second-hand tobacco smoke by improving their knowledge, attitude and behavior post intervention and estimating salivary cotinine levels as markers of SHS exposure.

In the present study, when the mean salivary cotinine levels were compared pre- and postintervention, we found that although the mean postintervention salivary cotinine levels increased in both the experimental and control groups, the increase was less in the experimental group than in the control group. Although the difference was not statistically significant, it can be considered clinically significant since salivary cotinine levels greater than 0.1 ng/ml (20) demonstrate exposure to SHS. An increase in the mean salivary cotinine level of 0.13 ng/ml in the control group definitely points toward a significant increase in the exposure to SHS. This finding is also significant because this increase in the mean salivary cotinine levels was noticed in one month in the control group without the 40-min health education intervention.

Despite an extensive search of all the databases, we could not find studies comparing salivary cotinine levels before and after a health educational intervention among adolescents. However, studies (26, 27) among adults have shown that the percentage of participants with measurable concentrations of salivary cotinine decreased considerably after intervention in the form of legislation.

In the present study, all 60 participants reported exposure to SHS at home at baseline. When we analysed the pre- and postintervention responses of the groups regarding exposure to SHS, we found that at the start of the study, although all participants had a smoker who lived with them, one month after the intervention, we found that the number of participants who had a smoker who lived with them had reduced to 8 in the experimental group and to 10 in the control group. Even the number of guests who smoked in their house was reduced in both groups. We also found that the number of people who smoked inside the house decreased after the intervention. This was a very promising finding. However, an interesting finding was that after the intervention, there was a statistically significant increase in the number of people smoking in front of children at home in the experimental group compared to the control group. This could be attributed to the fact that with increasing awareness about the effects of SHS, the children could have begun noticing smoking behaviour at home.

When we analysed the responses of the groups based on the comparison of baseline and final scores of the questionnaire, we found that in the knowledge domain, there was a statistically significant increase in the knowledge of the participants in 8 out of the 10 items in the experimental group. Similar findings were reported by Raji et al. (28) in a study on Nigerian adolescents.

In the attitude domain, we found that out of the 5 items, 3 items showed an improvement in the experimental group compared to only one item in the control group. In the avoidance behavior domain, all 5 items showed improvement in the experimental group compared to only 2 items in the control group. A dearth of published studies pertaining to intervention programs targeted at children to reduce their exposure to SHS compelled us to discuss our findings only with the limited evidence available in the literature. Similar findings were reported by Raji et al. (28), who reported a postintervention increase in the percentage of adolescents who reported that they would leave a place where cigarettes were being smoked. In the self - efficacy of avoidance domain, all 5 items showed a statistically significant improvement in the experimental group compared to the control group.

## 5. Conclusions

The present study has been shown to be effective in improving the knowledge, attitude and avoidance behavior of adolescents toward exposure to secondhand smoke. When we compared the overall pre- and postintervention domain scores, we found that all the domains showed improvement in the experimental group compared to the control group. When the mean salivary cotinine levels were compared pre- and postintervention, we found that although the mean postintervention salivary cotinine levels increased in both the experimental and control groups, the increase was less in the experimental group than in the control group. This study shows that the educational intervention program is effective in improving the knowledge, attitude, avoidance behavior and self-efficacy of avoidance among adolescents toward exposure to secondhand smoke, and the findings of this randomized controlled trial can be generalized to the adolescent population worldwide.

## 6. Recommendations

Smoke-free air is a fundamental human right, and everyone has the right to breathe clean air. Children, unlike adults, are unable to control their exposure to tobacco smoke. They are compelled to live in the environment that has been established for them by adults. Although tobacco control measures that educate the public about the dangers of smoking around children are crucial in making houses smoke-free, this study has also shown the positive effect of targeting children themselves. Educating children about the harms of secondhand tobacco smoke would make them aware of the environmental tobacco smoke around them and empower them to try and avoid exposure to SHS. It could also motivate them to never indulge in tobacco habits in the future. A trickle effect of educating children could be that they could also play a role in educating their parents and motivating them to stop tobacco smoking at home. A dedicated hour every week incorporated into the school curriculum to educate children about the ill effects of tobacco would help them develop a healthy attitude and avoid exposure to SHS. The findings can also be applied in designing educational programs or policies contributing to broader efforts in public health and tobacco control. This could go a long way in not only bringing about awareness and improved health but also developing a healthy attitude and lifestyle in our future generation, converting our dream of a tobacco-free world into reality.

## 7. Limitations

Although salivary cotinine is one of the most reliable markers for SHS exposure, it has a half-life of 16 h and can be detected for a limited time period in saliva, which could be a limitation. This study analysed the baseline and postintervention findings over a period of 30 days, which can be considered a short-term effect. Continued analysis of the effects of periodic “Tobacco-free” intervention for a better understanding of the long-term impact on SHS exposure as well as knowledge, attitude and behaviour needs to be carried out in this group of children.

## Data Availability

The raw data supporting the conclusions of this article will be made available by the authors, without undue reservation.
